# A three-dimensional multiscale model for the prediction of thrombus growth under flow with single-platelet resolution

**DOI:** 10.1371/journal.pcbi.1009850

**Published:** 2022-01-28

**Authors:** Kaushik N. Shankar, Yiyuan Zhang, Talid Sinno, Scott L. Diamond

**Affiliations:** Department of Chemical and Biomolecular Engineering, Institute for Medicine and Engineering, University of Pennsylvania, Philadelphia, Pennsylvania, United States of America; University of Pittsburgh, UNITED STATES

## Abstract

Modeling thrombus growth in pathological flows allows evaluation of risk under patient-specific pharmacological, hematological, and hemodynamical conditions. We have developed a 3D multiscale framework for the prediction of thrombus growth under flow on a spatially resolved surface presenting collagen and tissue factor (TF). The multiscale framework is composed of four coupled modules: a Neural Network (NN) that accounts for platelet signaling, a Lattice Kinetic Monte Carlo (LKMC) simulation for tracking platelet positions, a Finite Volume Method (FVM) simulator for solving convection-diffusion-reaction equations describing agonist release and transport, and a Lattice Boltzmann (LB) flow solver for computing the blood flow field over the growing thrombus. A reduced model of the coagulation cascade was embedded into the framework to account for TF-driven thrombin production. The 3D model was first tested against in vitro microfluidics experiments of whole blood perfusion with various antiplatelet agents targeting COX-1, P_2_Y_1_, or the IP receptor. The model was able to accurately capture the evolution and morphology of the growing thrombus. Certain problems of 2D models for thrombus growth (artifactual dendritic growth) were naturally avoided with realistic trajectories of platelets in 3D flow. The generalizability of the 3D multiscale solver enabled simulations of important clinical situations, such as cylindrical blood vessels and acute flow narrowing (stenosis). Enhanced platelet-platelet bonding at pathologically high shear rates (e.g., von Willebrand factor unfolding) was required for accurately describing thrombus growth in stenotic flows. Overall, the approach allows consideration of patient-specific platelet signaling and vascular geometry for the prediction of thrombotic episodes.

## Author summary

The excessive formation of blood clots under flow within the circulatory system (thrombosis) is known to initiate heart attacks and strokes. Therefore, obtaining insights into the formation and progression of these clots will be useful in evaluating pharmacological options. To this end, we have developed a 3D computational model that tracks the growth of a blood clot under flow from initial platelet deposition to full vessel occlusion in the presence of soluble platelet agonists. We first validated the model against experimental predictions of blood clots formed in vitro. Due to the construction of the model in 3D, we were able to carry out simulations of clot formation under important clinical situations, namely cylindrical blood vessels and acute flow narrowings (stenoses). To our knowledge, our model is the first of its kind that can account for patient-specific platelet phenotypes to perform robust 3D simulations of thrombus growth in geometries of clinical relevance.

## Introduction

During atherosclerotic plaque rupture that triggers thrombus growth, platelets respond to a combination of stimuli from multiple agonists, such as exposure to collagen and platelet-released ADP and thromboxane (TXA_2_). Additionally, tissue factor (TF) at the site of injury leads to the production of thrombin, another potent platelet agonist. These stimuli drive signaling pathways within platelets that elevate intracellular calcium, activate β_1_ and β_3_ integrins, and release α and dense granules. Increased platelet activity leads to further platelet recruitment and activation, resulting in a growing aggregate of platelets that obstructs blood flow. Excessive platelet deposition and aggregation due to platelet hyperactivity at the site of plaque rupture within the circulatory system (thrombosis) is known to initiate heart attacks and strokes. Quantifying the dynamics of thrombus growth and response to drug treatments would be an essential diagnostic tool for the evaluation of pharmacological options.

Thrombus growth is an extremely complex phenomenon that occurs over multiple length scales in the presence of spatial gradients within and around a clot, all of which underlie thrombus structure and function [1]. At the molecular level, there exist several cell signal transduction pathways, enzyme-substrate kinetics, and receptor activation processes [2]. At the resolution of a single platelet (~1μm), platelet-platelet adhesion and integrin activation lead to clot growth that affect and are in turn affected by the prevailing hemodynamics at the scale of a typical blood vessel (~1mm) [3]. At the mesoscale, within the clot boundary, soluble platelet agonist transport is diffusion-dominated, while bulk flow leads to dilution of these soluble species [4]. Therefore, computational modeling of thrombus growth provides an especially ripe opportunity to leverage tools from multiscale modeling and machine learning that can be validated against experimental observations and then be used to infer the dynamics of thrombus growth in situations of clinical relevance [5].

Numerous computational models of thrombus growth under flow have been developed in the literature [6–17]. While several continuum models of thrombus growth have been developed in 3D, single-platelet resolution models that are necessary to account for the stochastic thrombus morphologies have largely been restricted to 2D representations. Recognizing that the physics of hemodynamic flow and thrombus growth is inherently 3D, we have extended a multiscale model of platelet aggregation under flow in a 2D rectangular domain to solve the problem on a generalized 3D domain [11,16]. The model utilizes a neural network (NN) trained using calcium traces obtained for all single and pairwise combinations of six agonists at low, medium and high concentrations: ADP, thrombin, GSNO (NO donor), and mimetics for collagen, thromboxane, and prostacyclin, which were used to quantify P_2_Y_1_/P_2_Y_12_⁠, PAR-1/PAR-4, guanylate cyclase, GPVI, TP, and IP receptor signaling, respectively [18]. Platelet diffusive and convective motion and binding/unbinding are tracked by the lattice kinetic Monte Carlo (LKMC) method. Additionally, the model utilizes a velocity field solved using the lattice Boltzmann (LB) method and agonist concentration profiles obtained by finite volume method (FVM) solution of convection–diffusion-reaction equations. Furthermore, we have included a reduced model of the coagulation cascade that accounts for the concentrations of key coagulation proteins [19]. Thrombin concentration from the reduced model, along with concentrations of other agonist species from FVM was used as an input to the NN to determine platelet activation states. We have also included a coarse-grained shear-sensitive platelet adhesion function that characterizes the different adhesion receptors that are involved in thrombus growth.

## Materials and methods

**Fig 1:**
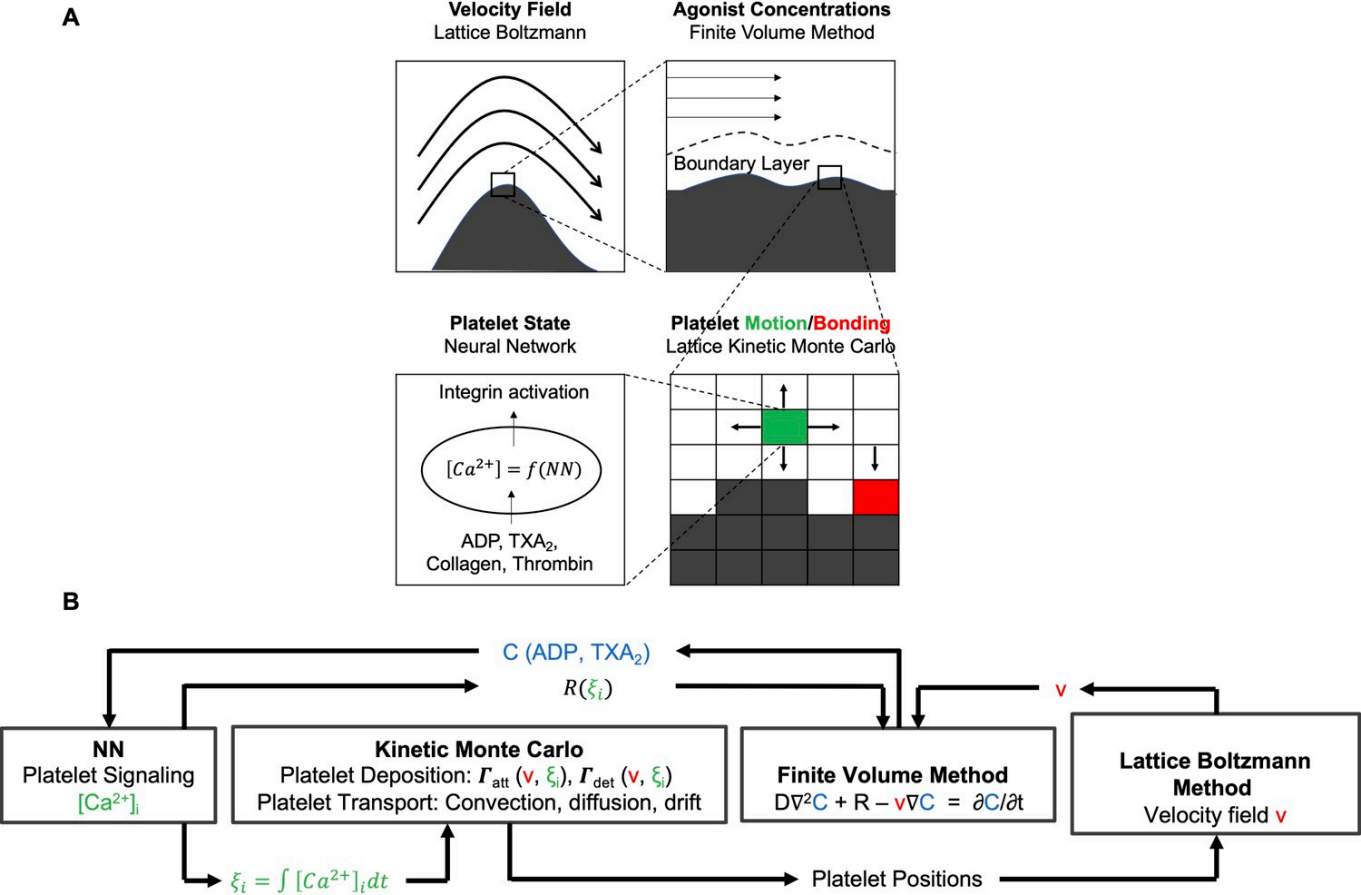
Multiscale model for thrombus growth under flow. The multiscale simulation of thrombus growth under flow required simultaneous solutions of the instantaneous velocity field over a complex and evolving platelet boundary by LB, concentration fields of ADP and TXA_2_ by FVM, individual intracellular platelet state ([Ca]_i_) and release reactions (R) for ADP and TXA_2_ by NN, and all platelet positions and adhesion/detachment by LKMC.

The multiscale framework is an extension of a 2D model from previous work to a fully spatially resolved 3D model and consists of four modules: neural network (NN), lattice kinetic Monte Carlo (LKMC), lattice Boltzmann (LB), and finite volume method (FVM) [*11*,*16*]. The NN module uses multicomponent agonist exposure data to determine a unique patient-specific intra-platelet calcium mobilization, which is then employed to determine the extent of integrin activation and adhesiveness of each platelet. Within the LKMC module, a rate database of all possible events is constructed, which in this case are platelet motion and binding events. In the LKMC simulation, the domain is discretized into a uniform simple cubic lattice upon which platelets are placed. Subsequent platelet motion or binding events are carried out at these lattice points. The rates of attachment and detachment of platelets to the reactive patch and/or to each other are a function of the activation state of the platelets (NN module) and the local shear rate around the platelets (LB module). The LB module solves indirectly the equations which describe the blood flow velocity in the domain. Here, we use the open-source LB solver Palabos [*20*]. The FVM module tracks local agonist concentrations (ADP and TXA_2_) by solving the convection-diffusion-reaction equation for species transport. The OpenFOAM software for scalar transport is used for the FVM module [*21*]. The Multiscale Universal Interface (MUI) is used to facilitate the exchange of information between individual modules of the multiscale framework [*22*]. A schematic of the multiscale simulation framework depicting the coupling between individual modules is provided in Fig *1*.

In our simulations, blood is assumed to be an incompressible Newtonian fluid where the governing equations for describing the velocity and pressure fields are:

$$\rho \left(\frac{\partial v}{\partial t}+v\cdot \nabla v\right)=-\nabla P+\mu \nabla^{2}v,$$ (1)

and

∇·v=0, (2)

where *ρ*, *μ*, **v**, *and P* are the blood density, blood viscosity, flow velocity vector field, and pressure field, respectively. This velocity field is utilized by LKMC to determine the local convective rate of platelet motion given by:

$$\Gamma_{C}=\frac{v\cdot e_{i}}{h_{LKMC}},$$ (3)

where **v** is the local velocity vector, ***e***_i_ represents the unit vector along a particular lattice direction, and h_LKMC_ is the LKMC lattice spacing.

The diffusive rate of platelet motion is given by:

$$\Gamma_{D}=\frac{D_{platelet}}{h_{LKMC}^{2}},$$ (4)

where *D*_*platelet*_ is the platelet diffusion coefficient.

The total rate of platelet motion is determined as the sum of the convective and diffusive rates given by:

$$\Gamma_{motion}=\Gamma_{C}+\Gamma_{D}.$$ (5)

The presence of red blood cells leads to platelet migration and increased platelet concentration near the walls. This effect was accounted for by imposing a nonuniform platelet concentration at the inlet of the simulation domain, creating a physiologically consistent wall excess of platelets [23].

The NN module accounts for exposure to collagen, ADP, TXA_2_, thrombin, GSNO, and iloprost, and computes the time-dependent calcium concentration of each platelet. The activation state for platelet *i* at any time *t*, *ξ*_*i*_*(t)*, is defined as the time-integral of calcium concentration above the basal level ([Ca^2+^(0)] = 100nM):

$$\xi_{i}(t)=\int_{0}^{t}(\left[Ca^{2+}(t)]-[Ca^{2+}(0)\right])\cdot dt.$$ (6)

The recent-history activation state of platelet *i*, *ξ*_*Δt*,*i*_*(t)*, is determined as the integrated calcium level between the current time *t* and the previous time *t–Δt*:

$$\xi_{\Delta t,i}(t)=\int_{t-\Delta t}^{t}(\left[Ca^{2+}(t)]-[Ca^{2+}(0)\right])\cdot dt.$$ (7)

The Hill function is used to normalize the cumulative and recent-history calcium integrals between the basal and maximal levels of activation *α*_*min*_ and *α*_*max*_ to determine a time-dependent extent of inside-out signaling and integrin activation, *F*, where:

$$F(\theta_{i})=\alpha_{min}+(\alpha_{max}-\alpha_{min})\frac{\theta_{i}^{n}}{\theta_{i}^{n}+\theta_{50}^{n}};\theta_{i}=\xi_{i},\xi_{\Delta t,i}.$$ (8)

Next, we use a phenomenological model to correlate the enhancement of platelet adhesion rate, *E*, with the local shear rate *γ*. This model accounts for the platelet adhesion mechanisms over a wide range of shear rates, such as GPIbα/VWF, GPVI/collagen, α_2_β_1_/collagen, α_IIb_β_3_/fibrinogen, and α_IIb_β_3_/VWF [3]. The enhancement factor is parametrized as a function of the local shear rate to capture the different receptors that are active at different shear regimes, and is given by:

$$E(\gamma )=\{\begin{array}{ccc} 1 & , & \gamma \leq 3000\;s^{-1}\\ 1+19\cdot \frac{\gamma -3000}{5000} & , & 3000<\gamma \leq 8000\;s^{-1}\\ 20 & , & \gamma >8000\;s^{-1} \end{array}.$$ (9)

The 20-fold enhancement in adhesion above a shear rate of 8000s^-1^ is consistent with the increase in number of exposed ligands on the VWF A1 domain as result of a conformational change from a globular to a stretched conformation at these pathological shear rates [24–26].

The effective rate of platelet attachment to the reactive collagen surface is modeled as:

$$\Gamma_{att}^{collagen}=k_{att}^{collagen}\cdot F(\xi_{i})\cdot F(\xi_{\Delta t,i})\cdot E(\gamma_{i}),$$ (10)

where $k_{att}^{collagen}$ is the attachment rate of a fully activated platelet and accounts for receptor and ligand copy numbers and single bond kinetics, *F(ξ*_*i*_*)F(ξ*_*Δt*,*i*_*)* represents the extent of inside-out signaling for integrin activation, and *E(γ*_*i*_*)* represents the amplification in attachment rate due to VWF, where *γ*_*i*_ is the local shear rate around platelet *i*.

The effective attachment rate between platelet *i* and platelet *j* was modeled using the geometric mean of integrin activation:

$$\Gamma_{att}^{platelet}=k_{att}^{platelet}\cdot {\left(F(\xi_{i})\cdot F(\xi_{j})\cdot F(\xi_{\Delta t,i})\cdot F(\xi_{\Delta t,j})\cdot E(\gamma_{i})\cdot E(\gamma_{j})\right)}^{\frac{1}{2}}.$$ (11)

The shear-dependent breakage of receptor-ligand bonds is characterized using a parametrized function *G*, to determine the enhancement of platelet detachment rate with shear:

$$G(\gamma )=\left\{ \begin{array}{ccc} \exp\left(\frac{\gamma }{\gamma_{c}}\right) & , & \gamma \leq 1000\;s^{-1}\\ \exp\left(\frac{1000}{\gamma_{c}}\right)\cdot \exp\left(\frac{\gamma }{\gamma_{c}^{\prime }}\right) & , & \gamma >1000\;s^{-1} \end{array}\right.,$$ (12)

where *γ*_*c*_ and *γ*_*c*_*’* are the characteristic shear rates required to initiate bond breakage. The form of *G* is modeled using the Bell exponential to describe the shear-dependent breakage of receptor-ligand bonds [27].

The detachment rate between a platelet and the reactive surface is modeled as:

$$\Gamma_{det}^{collagen}=k_{det}^{collagen}\cdot F^{-1}(\xi_{i})\cdot F^{-1}(\xi_{\Delta t,i})\cdot E^{-1}(\gamma_{i})\cdot G(\gamma_{i}),$$ (13)

where *F*^*-1*^*(ξ*_*i*_*)F*^*-1*^*(ξ*_*Δt*,*i*_*)E*^*-1*^*(γ*_*i*_*)* is a metric of the number of bonds that must be broken, and *G(γ*_*i*_*)* represents the acceleration of bond breakage due to shear forces.

Similarly, the detachment rate between platelet *i* and platelet *j* is modeled as:

$$\Gamma_{det}^{platelet}=k_{det}^{platelet}{\left(F(\xi_{i})\cdot F(\xi_{j})\cdot F(\xi_{\Delta t,i})\cdot F(\xi_{\Delta t,j})\cdot E(\gamma_{i})\cdot E(\gamma_{j})\right)}^{-\frac{1}{2}}\cdot {\left(G(\gamma_{i})\cdot G(\gamma_{j})\right)}^{\frac{1}{2}}.$$ (14)

Agonist concentration fields *C*_*j*_*(x*,*y*,*z*,*t)* (where *j* = ADP, TXA_2_) are determined using the FVM solution to convection-diffusion-reaction equations:

$$\frac{\partial C_{j}}{\partial t}+v\cdot \nabla C_{j}=D_{j}\nabla^{2}C_{j}+R_{j},$$ (15)

where *D*_*j*_ and *R*_*j*_ (j = ADP, TXA_2_) are the diffusion coefficient and the volumetric release rate respectively, and **v** is the local flow velocity. The release of ADP and TXA_2_ is assumed to occur only when the cumulative activation state of the platelet becomes larger than the critical threshold *ξ*_*crit*_. The rate of release of ADP and TXA_2_ is modeled as:

$$R_{j}=\frac{M_{j}}{\tau_{j}}\cdot \exp(\frac{t_{\mathrm{release}}-t}{\tau_{j}}),$$ (16)

where *M*_*j*_ is the total amount of releasable ADP or TXA_2_ in a platelet and τ_*j*_ is the characteristic time constant of release. If a platelet became sufficiently activated but was not bound, it was still allowed to be a moving source of ADP and TXA_2_. Detailed information about each module of the multiscale model has been provided in S1 Appendix, along with a list of all the parameters used.

To account for the effect of wall-derived TF, a reduced model of the coagulation cascade developed by Chen and Diamond is used to determine the concentration of thrombin within the 15-micron fibrin-rich core of the clot [19]. The ~15μm thickness of the fibrin layer has been measured experimentally in a side-view chamber (Fig S5 in [28]) and by calibrated epifluorescence (Fig 7 in [29]). Since fibrin is the non-diffusible end-product of coagulation, we consider this fibrin layer to be the zone of coagulation assembly.

The model considers a simplified reaction network consisting of 6 reactive species that included extrinsic tenase/FIXase activity, intrinsic tenase activity, prothrombinase activity, feedback activation of FXIa by thrombin, fibrin generation, and thrombin binding to fibrin (see Fig A in S1 Appendix) using measured Michaelis-Menten kinetic parameters. Furthermore, the model makes use of a thin-film approximation for the fibrin-rich clot core (thickness 15μm) where the initial and prevailing concentrations of coagulation zymogens are set to be identical to those in flowing plasma. To account for transport limitations, effectiveness factors (actual rate / ideal rate in the absence of transport limitations) were introduced to scale the reaction rates. The effectiveness factors were obtained by fitting model predictions with direct experimental observations of F1.2 and TAT production [19]. Within the film, the species are assumed to be spatially well-mixed and only depend on time. This assumption allowed a significant reduction in the computational cost involved in simulating the full coagulation cascade consisting of ~50 partial differential equations to a simple model consisting only of 8 ordinary differential equations (ODEs). As an ODE model, the actual transport physics were mainly parameterized by the rate of thrombin elution from the clot, guided by experimental measurements of ~2-sec half-life of flash-activated albumin in a clot subjected to flow along its outer boundary. The 2-sec elution half-life for proteins was consistent with in-vivo mouse model measurements and human blood microfluidic measurements.

The thrombin concentration predicted by the reduced model is used as an input to the NN module to estimate intracellular calcium for platelets within the thin-film region (within 15μm normal to TF surface). Platelet activation due to thrombin is assumed to be present only within this thin-film region, and thrombin is not monitored outside this region. Furthermore, this thin-film region is assumed to be present from the start of the simulation. These assumptions are supported by experiments demonstrating that little thrombin leaks out of a clot under flow due to its sequestration by fibrin. Only when Gly-Pro-Arg-Pro is used to inhibit fibrin polymerization can thrombin be detected (as thrombin-antithrombin complex) in the clot effluent [30]. The kinetics of thrombin generation in the thin-film region are such that thrombin concentration does not become a significant factor in platelet activation until much of the thin-film region is occupied by platelets. More details about the model as well as the full list of rate constants and equations are given in S1 Appendix.

## Results

### Thrombus growth in a microfluidic device

**Fig 2:**
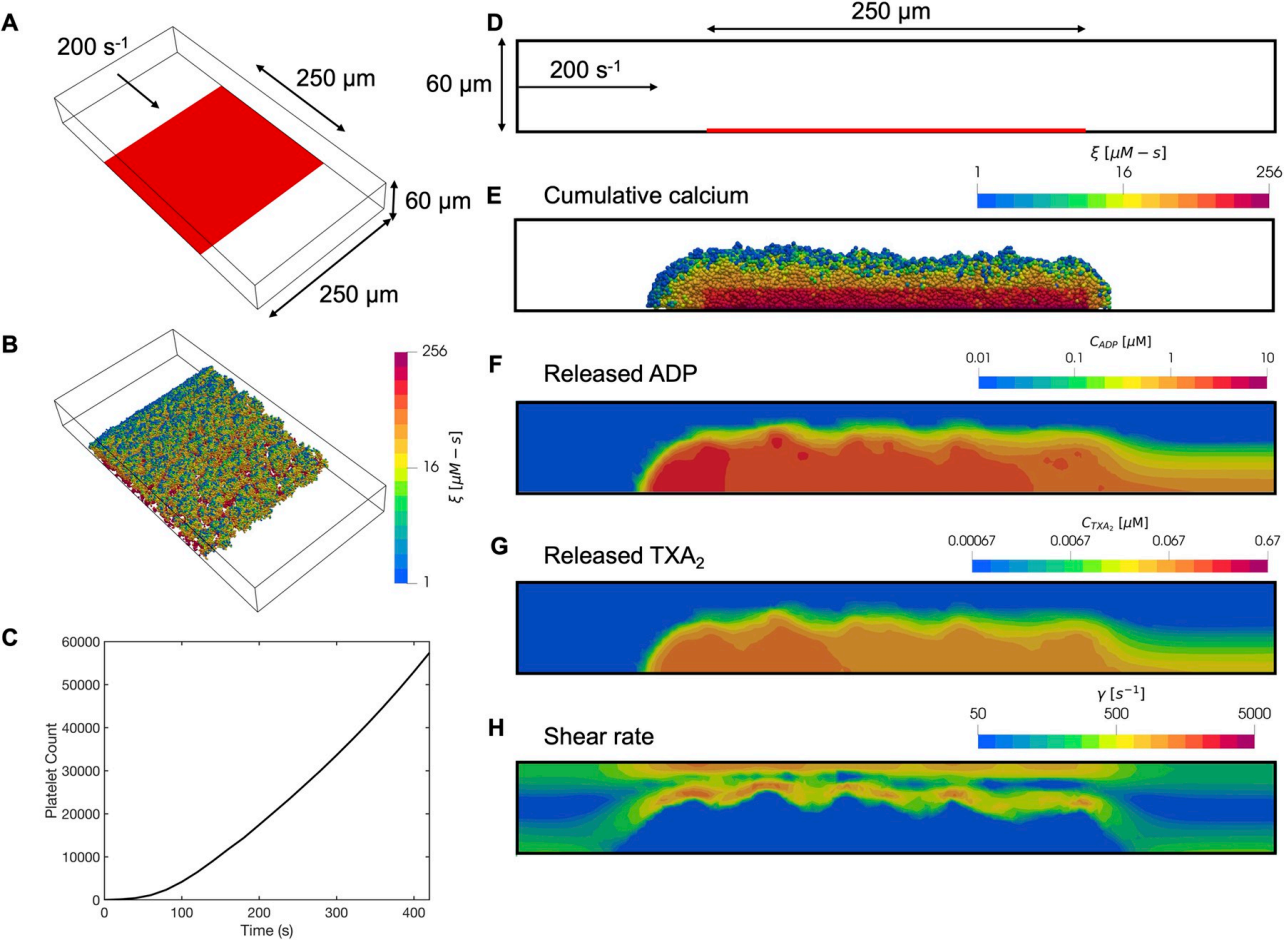
Simulation results of platelet aggregation under flow in the microfluidic device. Schematic of the microfluidic device geometry: (A) oblique view and (D) side view. Inlet flow: 200s^-1^; reactive surface containing collagen and TF: red bar. (B,E) Platelet activation (blue indicates inactivated and red, fully activated) and deposition after 400 seconds. (C) Dynamics of platelet aggregate count observed over time. Released (F) ADP and (G) TXA_2_, and (H) the local shear rate contours plotted along the center of the channel after 400 seconds.

First, we validated our multiscale model by comparing model predictions to in vitro experiments of whole blood perfusion in an 8-channel microfluidic device; see Figs 2A and 2D. Each channel is 250μm wide and 60μm high, with a 250μm x 250μm reactive surface containing collagen and TF representing an area of injury. The 3D multiscale model domain corresponds to a 500μm x 250μm x 60μm channel which includes this reactive surface at the boundary. At the inlet of the channel, a constant wall shear rate of 200s^-1^ was maintained over the entire course of the experiments and in the multiscale simulations.

The simulations captured individual platelets that deposited and aggregated on the reactive surface, their corresponding activation levels, the spatiotemporal distribution of ADP and TXA_2_, and the shear rate profile over the growing clot (Fig 2). During the course of the simulations, shear rates typically increased by an order of magnitude compared to the inlet shear rate as large platelet aggregates obstructed the channel. Platelets initially adhered only to the reactive surface. Upon significant calcium mobilization that reached the critical threshold *ξ*_*crit*_ after ~1 minute, platelets became sufficiently activated to release ADP and TXA_2_. The concentration profiles of ADP and TXA_2_ formed a boundary layer extending up to 10μm from the platelet deposit, which was sufficient to recruit platelets in the near-wall region. The released ADP and TXA_2_ worked synergistically with thrombin in the thin film to further strengthen platelet activation. Boundary layer concentrations of ADP and TXA_2_ were within the effective dynamic range (0.1–10 EC_50_). Platelets that were exposed to collagen and thrombin within the thin film were significantly more activated due to sustained calcium mobilization, whereas platelets that were stimulated only by ADP and TXA_2_ demonstrated much less activation. This gradient in platelet activation is consistent with in vivo mouse models that have shown the existence of a core/shell architecture with a highly activated P-selectin positive platelet core localized at the reactive surface that is surrounded by a shell of P-selectin negative platelets [31].

**Fig 3:**
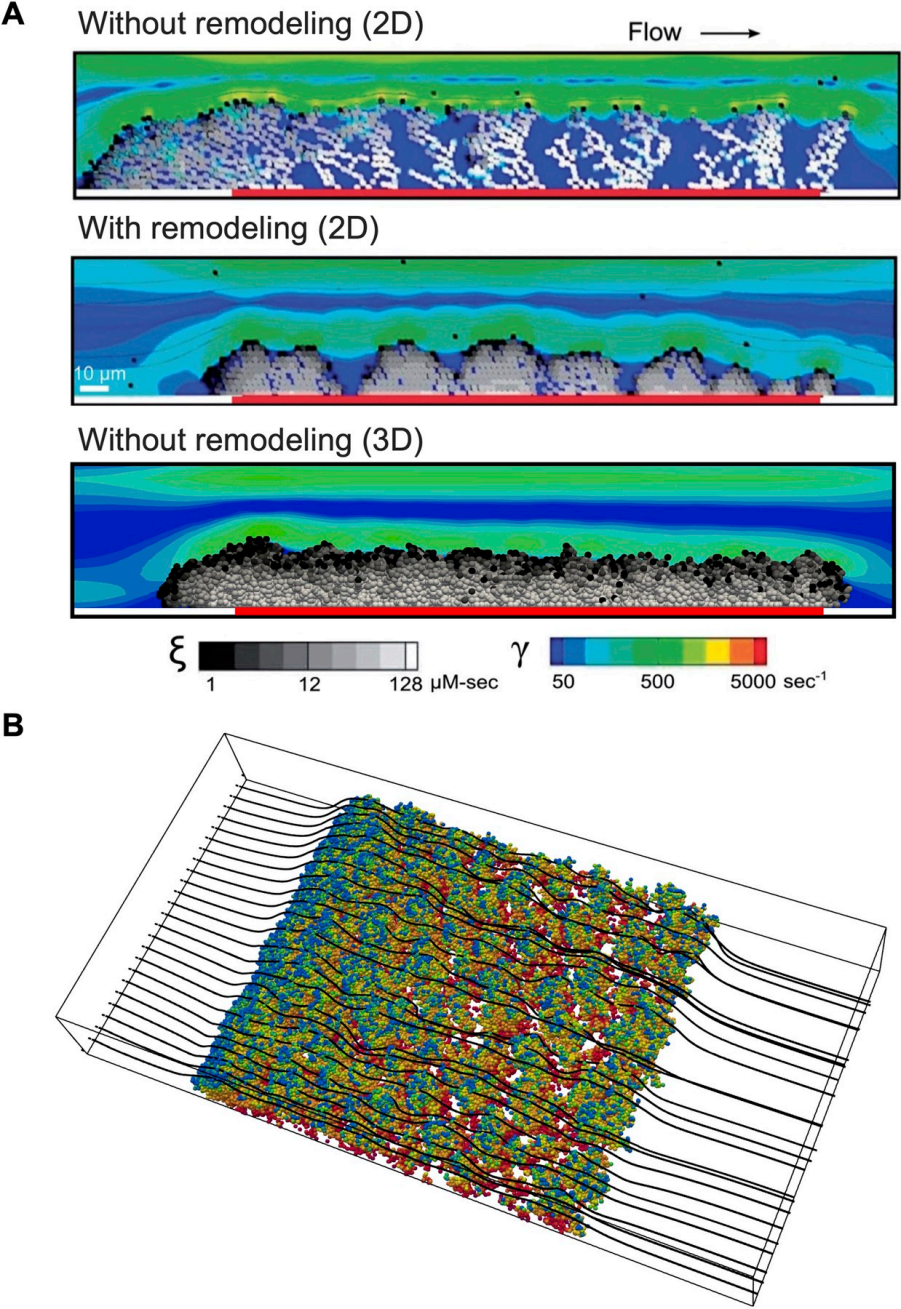
Comparison between prior 2D simulations and present 3D simulations. (A) Thrombus morphology observed in 2D simulations (top) showing highly dendritic aggregate growth. A clot remodeling scheme was required in 2D to obtain a realistic morphology (middle). Thrombus morphology observed in 3D without employing a remodeling scheme (bottom). (B) Thrombus morphology observed in present work in 3D. Dendritic aggregate formation is drastically reduced due to realistic platelet trajectories in 3D, as evidenced by flow streamlines (black lines) that show flow around aggregates.

The 3D simulations overcame artifacts that were present in prior 2D work where unrealistic and mechanically unstable dendritic clot morphologies formed due to blocking of flow by adhered platelets [16]. In the 2D simulations, this artifact was resolved by including a clot remodeling algorithm, where upon binding, platelets were allowed to search for a locally favored binding site that maximized the number of platelet-platelet contacts, thereby leading to denser packing (Fig 3A). In 3D these artifacts are naturally resolved without additional remodeling schemes because the 3D simulations allow for flow around the deposited platelet mass, as illustrated by flow streamlines around the platelet aggregates in Fig 3B. This key difference between 2D and 3D simulations highlights the value of 3D modeling of thrombus growth.

**Fig 4:**
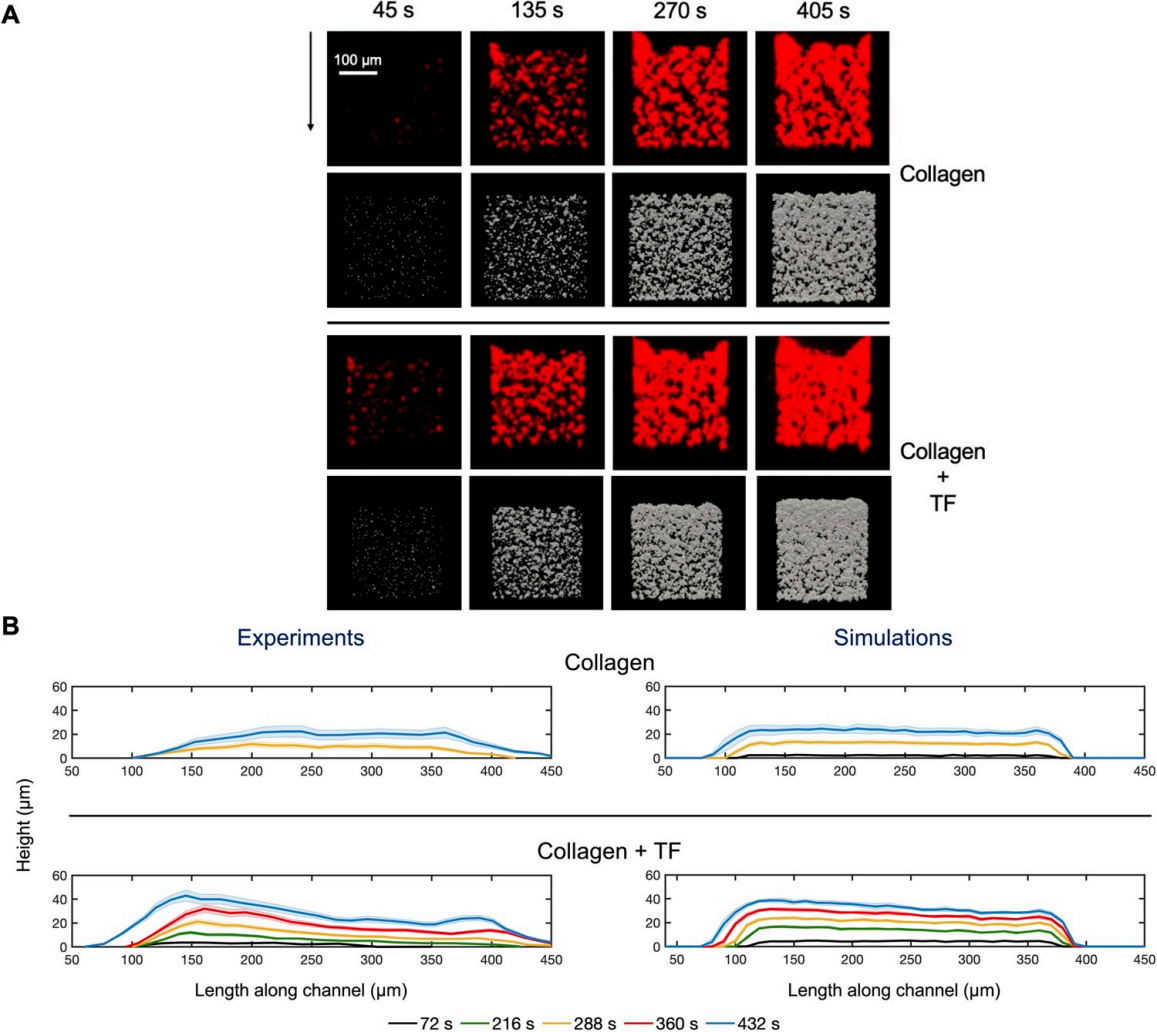
Comparison between model predictions and typical clot morphologies observed in microfluidic perfusion experiments. **(A)** Model predictions of deposited platelets (gray) agree well with fluorescent micrographs of thrombi observed over time in experiments (red). (B) Comparison between model predictions and clot heights observed in microfluidic perfusion experiments. Profiles represent average aggregate height determined using vertical line scans in the direction of flow over collagen or collagen with TF.

**Fig 5:**
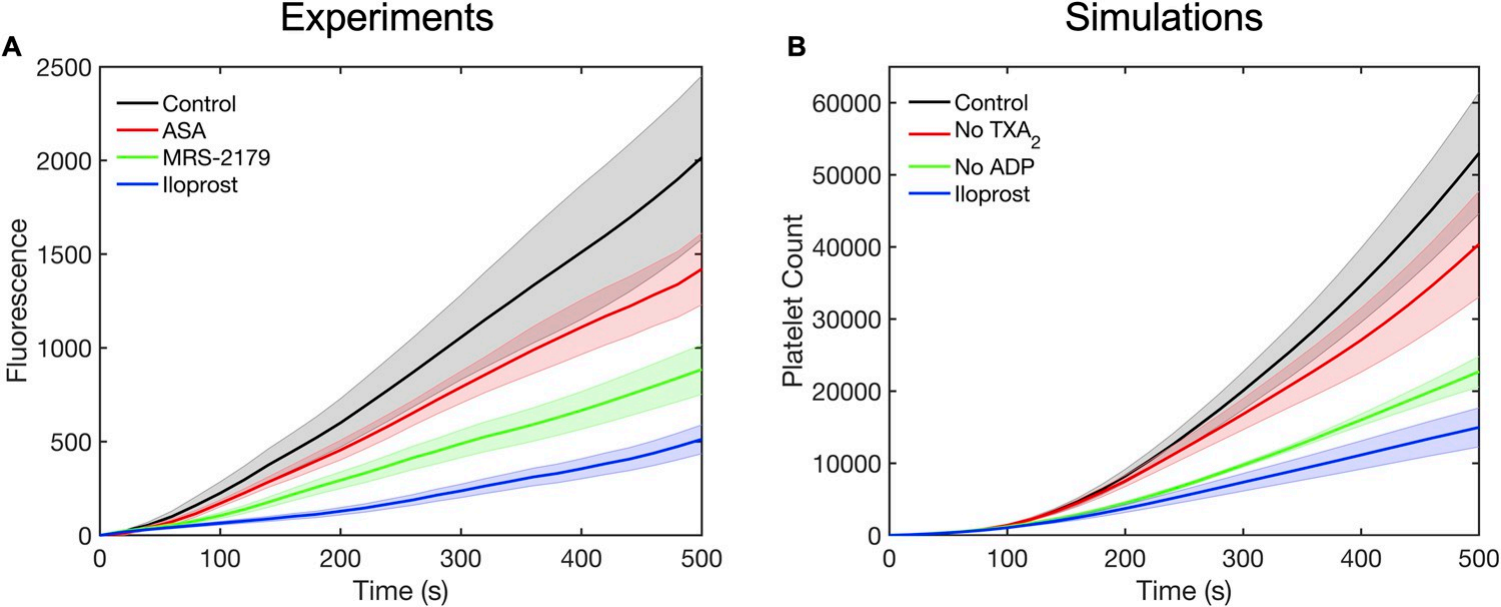
Comparison between thrombus growth dynamics observed in experiments and multiscale simulations under different agonist conditions. (A) Measured platelet deposition in the presence of aspirin, indomethacin, MRS 2179 and iloprost treatment by Flamm et al. and (B) corresponding multiscale simulations with no TXA_2_⁠, no ADP and iloprost stimulation.

The model was able to accurately capture the evolution and morphology of the thrombus initiated at the reactive surface. Model predictions of deposited platelets agreed well with fluorescent micrographs of thrombus formation over time observed in experiments for case studies with and without wall-derived TF (i.e., +/- thrombin), as shown in Fig 4A. Furthermore, profiles of average clot height along the length of the channel compared well against the experimental profiles that were generated by averaging line scans of fluorescent intensity drawn in the direction of flow (Fig 4B). Additionally, the function of pharmacological inhibitors was captured well by the model (Fig 5).

Thrombus growth favored the upstream end of the reactive surface, with platelets aggregating significantly even beyond the upstream borders, a feature consistent with experimental observations for case studies under both the presence and absence of TF [29]. The upstream thrombus extension is due to the presence of activated platelets at the leading edge of the clot which can recruit additional platelets even in the absence of the reactive collagen surface. However, at the downstream edge of the reactive surface, the simulations predicted an abrupt termination of platelet aggregation, in contrast with experiments that showed some clot extension beyond the edge of the reactive surface. A possible explanation for this discrepancy is the presence of collective platelet motion toward the downstream end of the clot due to fluid shear in the experiments [29]. Another possibility is that the platelet attachment rate at the trailing edge of the clot is more sensitive to the precise details of the flow and the role of red blood cells in enhancing the Brownian motion of the platelets.

In both experiments and simulations, the thrombus grew to a height of ~40μm after 400s in the presence of TF. However, when TF was absent, platelet deposition was significantly reduced to ~20μm. The inclusion of TF, and thereby, thrombin, led to significant differences in platelet activation when compared to other soluble agonists, with the observed platelet activation much higher under the presence of thrombin. Particularly, platelet signaling in response to thrombin displays unique properties of fast-on but slow-off due to PAR-1 and PAR-4 signaling dynamics captured in the NN training data.

Furthermore, the model was applied to study the sensitivity of platelet signaling and thrombus growth dynamics to drug treatments. The inhibition of agonists ADP and TXA_2_ was mimicked by setting their concentration to zero in the NN module. A comparison between the platelet deposition dynamics over collagen observed in experiments by Flamm et al. and the model predictions is shown in Fig 5, showing excellent agreement [11]. Inhibition of platelet signaling was most pronounced under IP receptor stimulation using the prostacyclin analog, iloprost. The removal of ADP by MRS-2179 treatment also showed a significant reduction in platelet deposition (~50% reduction). The removal of TXA_2_ using aspirin or indomethacin for COX-1 inhibition exhibited the least sensitivity.

### Thrombus growth in a straight cylindrical tube

**Fig 6:**
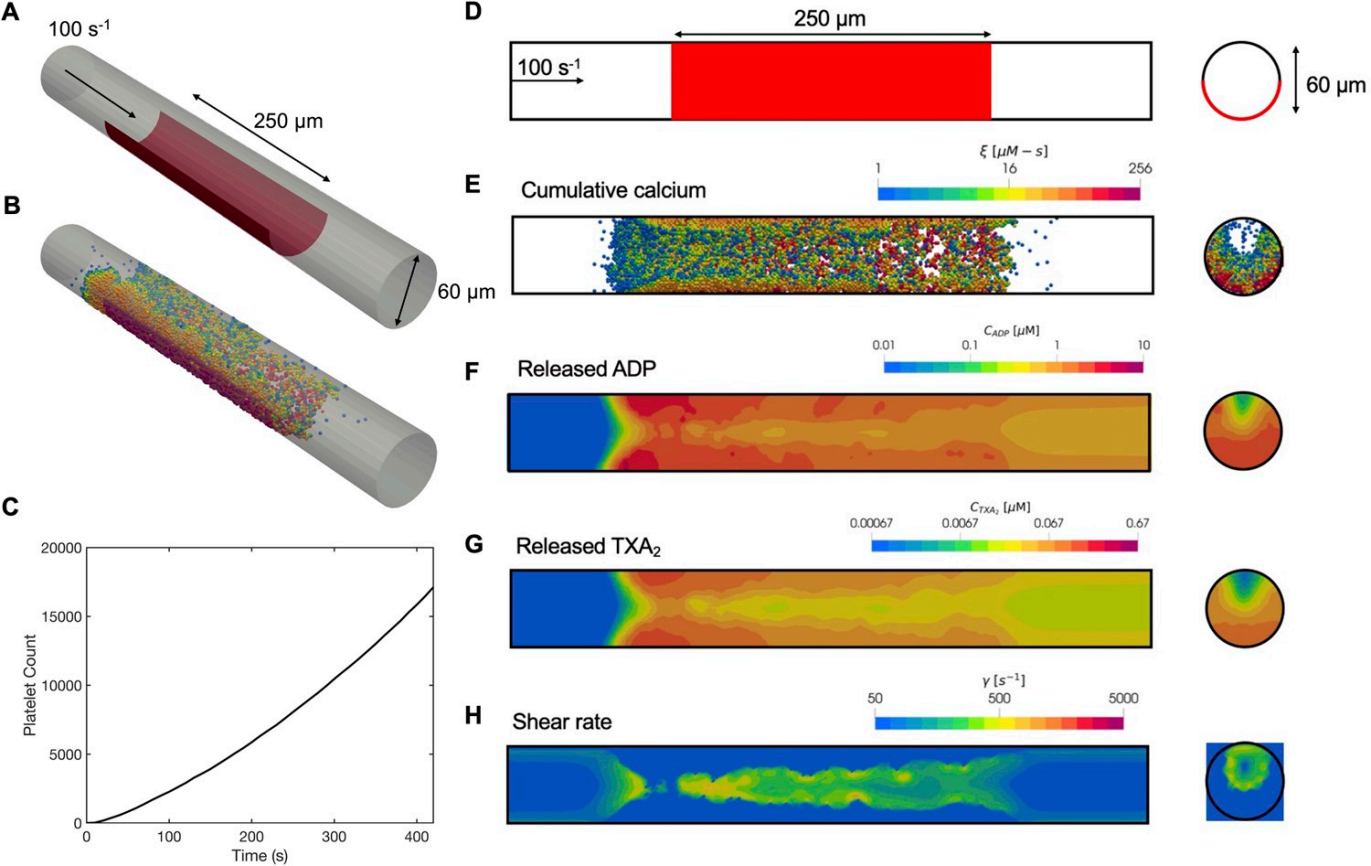
Simulation results of platelet aggregation under flow in a cylindrical tube. Schematic of the cylindrical tube geometry: (A) oblique view and (D) side view. Inlet flow: 100s^-1^; reactive surface containing collagen and TF: red bar. (B,E) Platelet activation (blue indicates inactivated and red, fully activated) and deposition after 400 seconds. (C) Dynamics of platelet aggregate count observed over time. Released (F) ADP and (G) TXA_2_, and (H) the local shear rate contours plotted along the center of the tube after 400 seconds.

The extension of the multiscale model from 2D to 3D enables the simulation of thrombus growth in a larger set of geometric domains. In this section, we consider a straight cylindrical tube that would represent a typical blood vessel; see Fig 6A and 6D. The tube is 500μm long and has a diameter of 60μm. A semicylindrical reactive surface containing collagen and TF representing an injury is located at the center of the tube. A constant wall shear rate of 100s^-1^ is maintained at the inlet, while a constant specified pressure was maintained at the outlet of the tube.

The multiscale predictions of platelet trajectories, ADP, TXA_2_, and shear profiles for the cylindrical tube geometry are presented in Fig 6. Similar to simulations of thrombus growth in our experimental microfluidic setup, we found that the number of aggregated platelets as a function of time exhibited an almost linear trend following an initial lag. The apparent lag time is physiologically driven and corresponds to the time when the first monolayer of platelets becomes sufficiently activated for dense granule release of ADP and TXA_2_. Platelet aggregates grew significantly beyond the borders of the reactive surface, both axially and radially. Thrombus growth occurred preferentially toward the upstream end of the reactive surface. Agonist concentration boundary layer thicknesses were similar to the microfluidic device simulations, extending ~10μm radially from the platelet deposit.

### Thrombus growth in a stenosis

**Fig 7:**
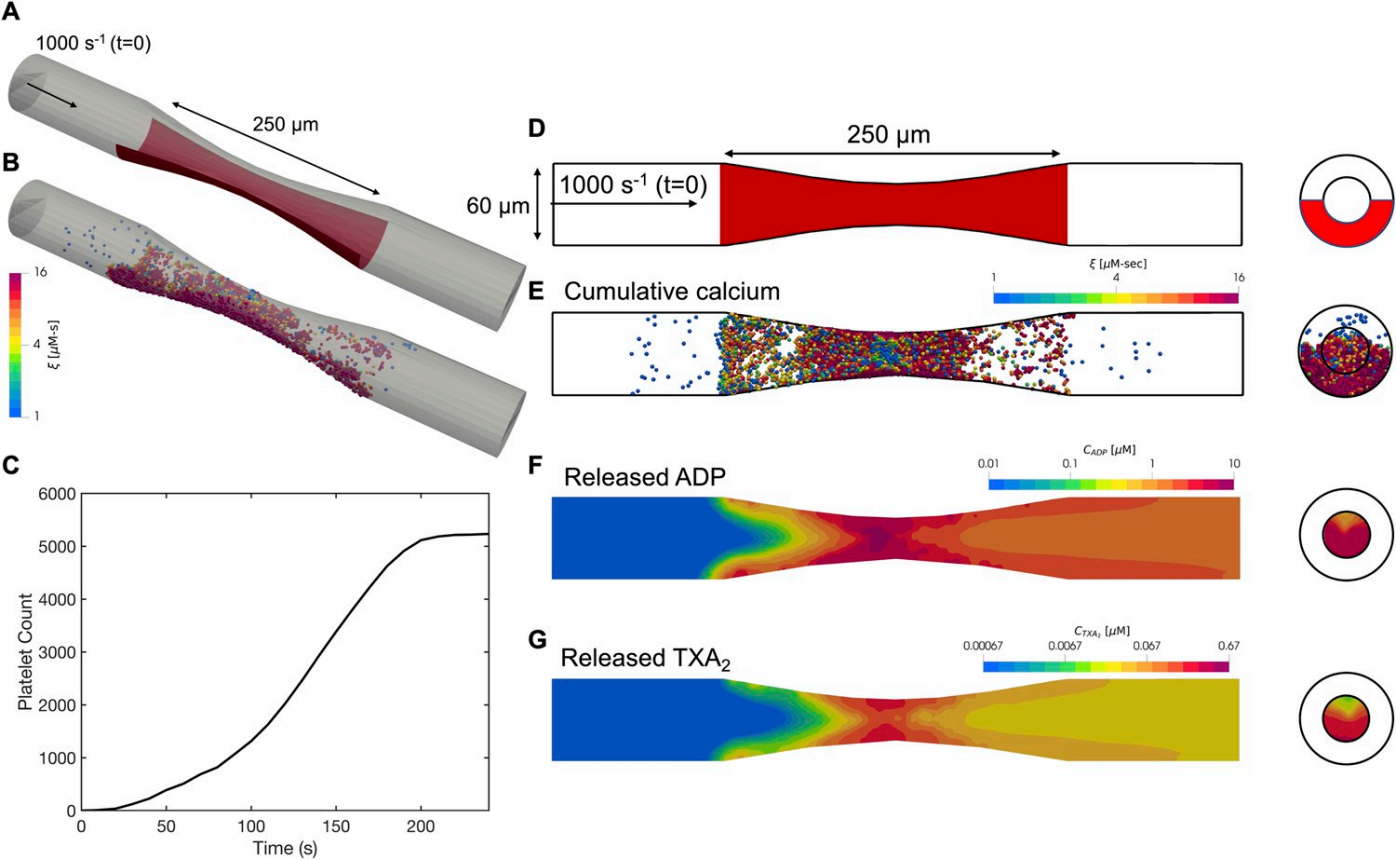
Simulation results of platelet aggregation under pathological shear rates in a stenosis at the time of vessel occlusion. Schematic of the stenotic geometry: (A) oblique view and (D) side view. Inlet and outlet of the stenosis maintained at a constant pressure drop across the length of the geometry that corresponded to an initial inlet wall shear rate of 1000s^-1^. Surface collagen: red bar. (B,E) Platelet activation (blue indicates inactivated and red, highly activated) and deposition at occlusion time. (C) Dynamics of platelet aggregate density observed over time. Released (F) ADP and (G) TXA_2_ contours plotted along the along the center of the tube at the time of occlusion.

During atherosclerotic plaque rupture, thrombus growth in high-shear environments created by the presence of acute flow narrowing (stenosis) is linked to heart attacks. Here, we present the results of model predictions of thrombus growth under flow in a cylindrical tube with a stenosis. We considered a cylindrical domain that is 500μm long and has an inlet diameter of 60μm. In the central 250μm of the tube, we imposed a 75% stenosis (75% reduction in lumen area), as shown in Fig 7A and 7D. The stenotic area is coated with a collagen surface that extends up to half the circumference. As platelets are deposited and the vessel becomes occluded, blood flow is diverted to other components of the circulatory system. This condition is fundamentally different from our experimental microfluidics condition used for validation of the model where a constant flow rate was maintained at the inlet. We therefore carried out these simulations under a constant specified pressure drop to better represent a physiologically relevant scenario, in which full occlusion may be reached. The inlet and outlet of the tube were maintained at a constant pressure drop that corresponded to an initial inlet wall shear rate of 1000s^-1^. This resulted in an initial pathological wall shear rate of 8000s^-1^ at the throat of the stenosis.

A snapshot of aggregated platelets, along with contours of ADP and TXA_2_ concentration profiles, is shown in Fig 7. These snapshots correspond to the time of occlusion, which we defined as the time at which the inlet volumetric flow rate dropped to less than 5% of its initial value. An interesting feature of our simulations under constant pressure drop is that the ADP and TXA_2_ concentration boundary layers at later time points closer to occlusion were much larger than those observed under constant flow. This was a direct result of the fact that flow reduces upon occlusion leading to lower convective transport of ADP and TXA_2_ away from the boundary layer. Therefore, the effects of ADP and TXA_2_ stimulation on platelet signaling and activation are particularly strong closer to occlusion, which has been observed experimentally to lead to a flow-dependent quorum sensing mechanism to help drive contraction [32].

**Fig 8:**
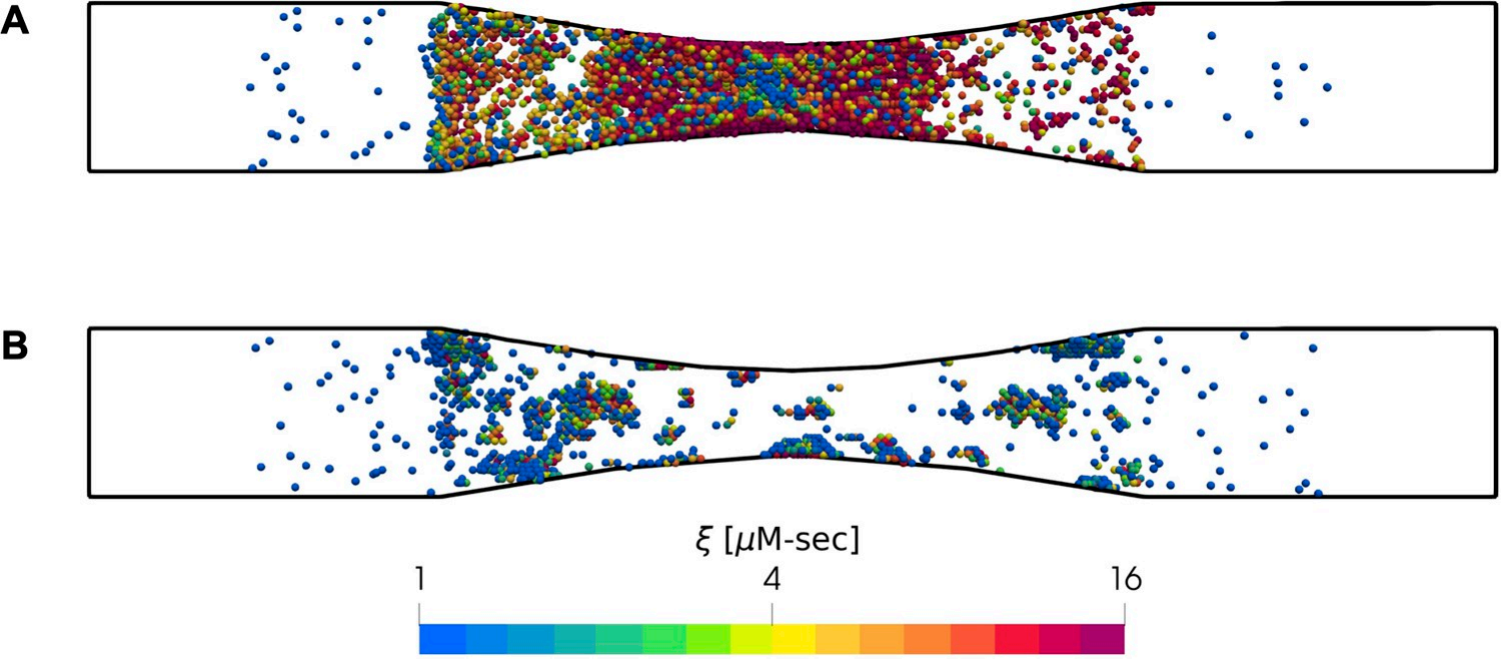
Effect of VWF mediated platelet capture at pathological shear rates. Simulation results of platelet activation (blue indicates inactivated and red, highly activated) in the (A) presence and (B) absence of enhanced clotting function due to VWF stretching at pathological shear rates > 3000s^-1^.

We observe that platelet aggregation under arterial shear occurs predominantly near the apex of the stenosis, where the observed shear rates are the highest. This observation is in contrast with thrombus growth under venous shear rates where platelets deposited preferentially toward the upstream end of the reactive surface. The presence of a stenosis creates a high-shear environment that causes VWF to undergo structural changes from a globular conformation to a stretched conformation. As described above, our platelet adhesion model (refer to Eq 9), includes an enhancement factor for the platelet adhesion rates, *E*, as a function of the local shear rate *γ*, at pathological shear rates greater than 3000s^-1^. There is a marked difference in the number and activation of aggregated platelets under the inclusion and exclusion of this enhancement as illustrated in Fig 8. This result highlights the necessity of VWF-conformational change for platelet aggregation at arterial shear rates. Under the absence of this elevated rate of platelet capture, platelets deposit at a much lower rate, and the high shear experienced by the platelets leads to their detachment from the clot mass. This is consistent with von Willebrand disease patients rarely presenting acute myocardial infarctions.

**Fig 9:**
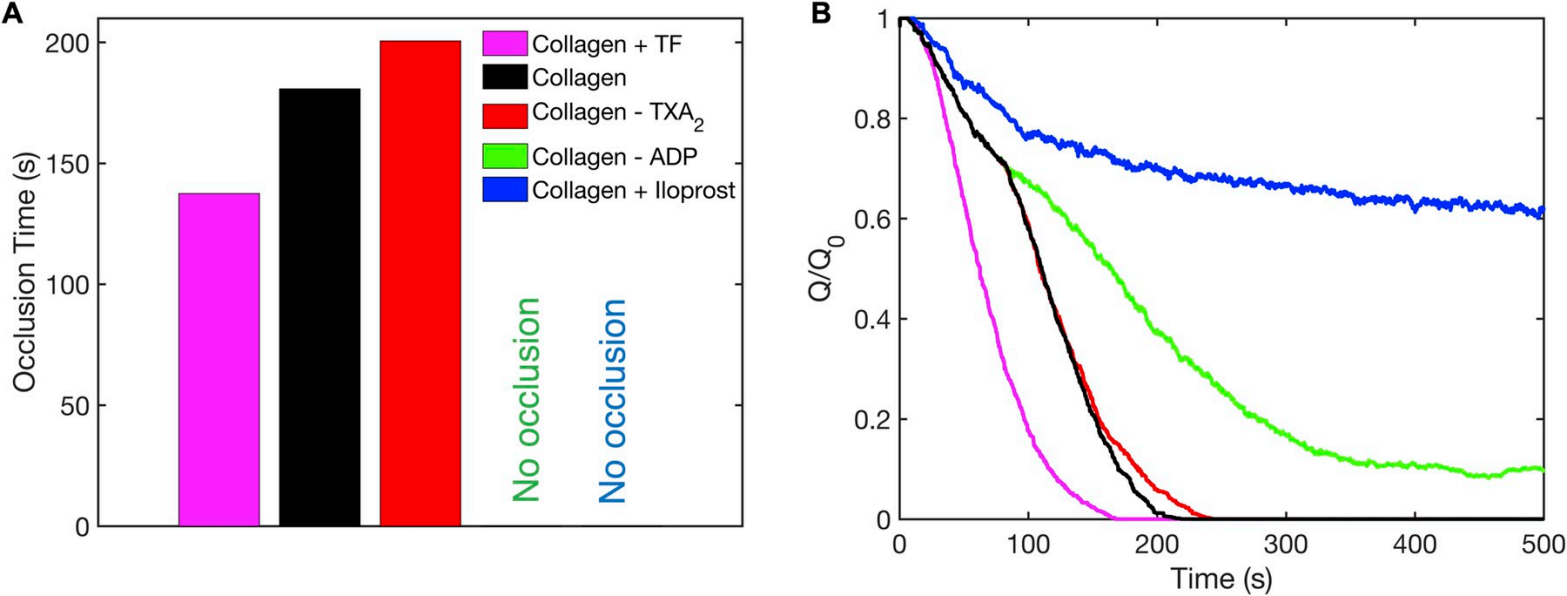
Platelet aggregation in a stenosis under agonist inhibition. (A) Observed occlusion times and (B) inlet volumetric flow rates (Q) versus time for different agonist conditions. The initial inlet flow rate (Q_0_) was set to 1.3μL/min, giving an initial inlet wall shear rate of 1000s^-1^.

To understand the effect of antiplatelet therapy on the rate of thrombus formation and vessel occlusion in arterial pathological shear environments, we carried out simulations similar to our drug response study in the microfluidic device. We present occlusion times and the change in inlet volumetric flow rate with time for each case in Fig 9. Compared to the control (platelet aggregation on collagen but no wall-derived TF), the inclusion of thrombin formation due to the presence of surface TF lowered the time to occlusion by ~20%. This modest decrease is consistent with the fact that anticoagulants are not typically used for coronary disease treatment. Inhibition of TXA_2_ by aspirin only had a mild effect on the occlusion time, increasing it by ~10% compared to the control. However, the inhibition of the more potent agonist, ADP, by MRS-2179 led to no occlusion. This is consistent with the notion that antiplatelet agents are essential tools to fight coronary thrombosis. Furthermore, the observed increase in occlusion time under TXA_2_ inhibition is consistent with in vitro observations of Li et al. under this range of shear rates, thereby validating our model predictions [33]. The complete inhibition of platelet activation by iloprost also led to no occlusion, highlighting the importance of exercise-induced release of endothelial prostacyclin. Platelet deposition reached a steady state for the last two conditions, as indicated by the inlet volumetric flow rate which fluctuated around a constant value (Fig 9B). The fluctuation arose due to platelet deposition followed by erosion.

**Fig 10:**
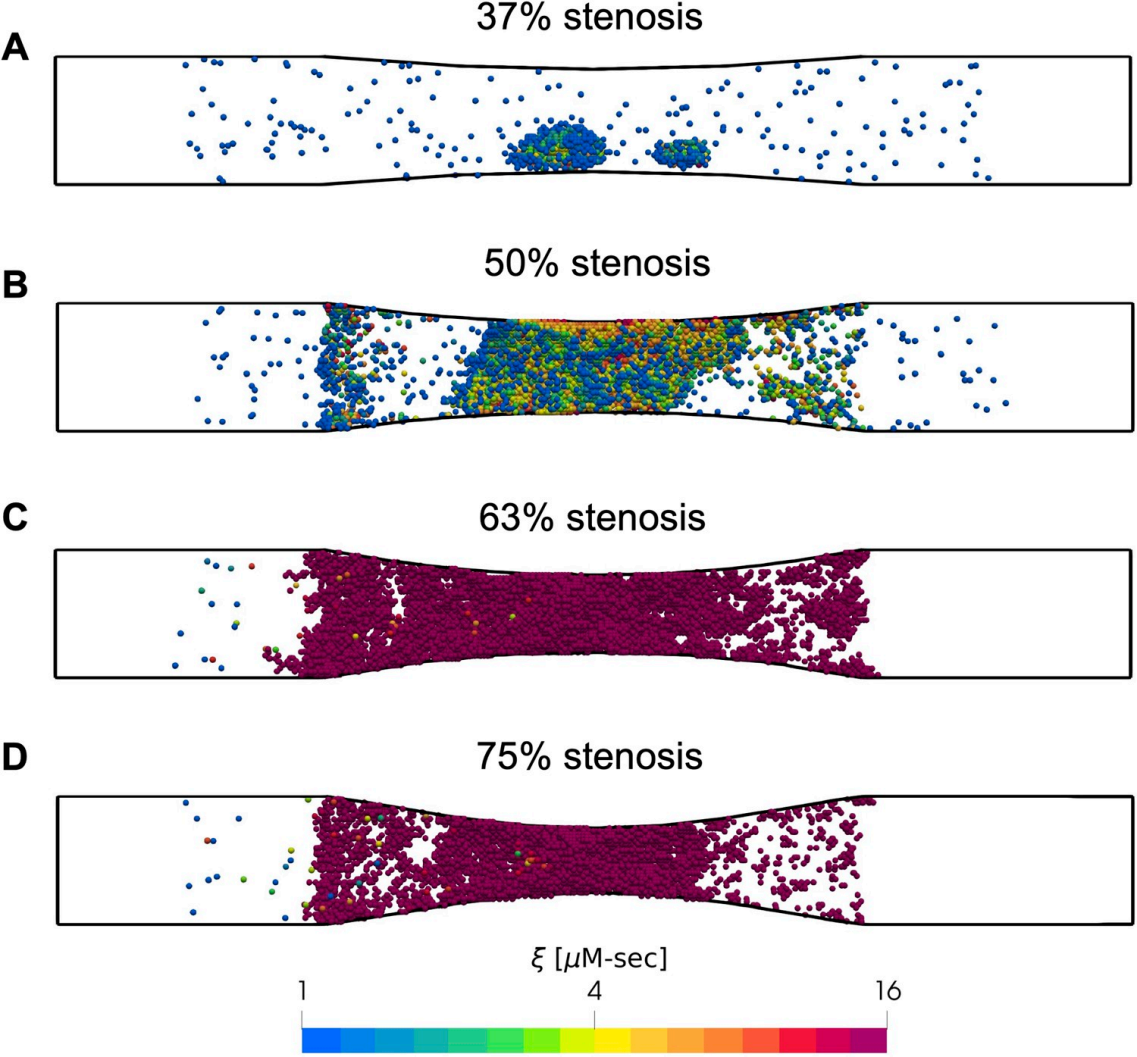
Effect of stenosis severity on platelet aggregation. Snapshots of platelets (blue indicates inactivated and red, highly activated) observed after 500 seconds are shown for stenoses with reductions in flow area of (A) 37%, (B) 50%, (C) 63%, and (D) 75%. Simulations were carried out under constant pressure drop that corresponded to an initial inlet wall shear rate of 1000s^-1^.

To investigate the role of stenosis severity on platelet aggregation, we carried out simulations of thrombus growth with stenoses corresponding to 37%, 50%, 63%, and 75% reductions in flow area. The inlets and outlets of the simulation domains were maintained at a constant pressure drop that corresponded to an initial inlet wall shear rate of 1000s^-1^. This value corresponded to initial wall shear rates of ~2000s^-1^, 3000s^-1^, 5000s^-1^ and 8000s^-1^ respectively at the stenosis apex. We present snapshots of platelet aggregates for each case in Fig 10. We observed little to no platelet aggregation in the 37% stenosis, where the shear rates were below the threshold shear rate for VWF-conformational change. In the 50% stenosis, platelet aggregation was delayed as the initial shear rates were around this threshold shear rate (3000s^-1^). As platelets deposited, the shear rate steadily rose well beyond the threshold, leading to significant platelet aggregation toward the later stages of the simulation. In the 63% and 75% stenoses, where the initial shear rates were well beyond the threshold value, the rate of platelet deposition was significantly enhanced, leading to complete occlusion of the domain within ~3 minutes, and significantly higher platelet activation. Taken together, these results are consistent with experimental observations by Li et al., who observed complete occlusion when the initial wall shear rate exceeded 4000s^-1^ [34].

## Discussion

The multiscale computational framework presented here extends prior efforts in the development of a robust model for thrombus growth under flow that accurately captures the prevalent aspects of platelet signaling, hemodynamics, soluble platelet agonists, and the stochastic nature of platelet motion and bonding. A key outcome of this work is the development and calibration of a 3D model validated against experiments of whole blood perfusion in microfluidic devices. The 3D simulations gave rise to more realistic predictions of thrombus morphologies, compared to the highly dendritic and mechanically unlikely structures that result in 2D simulations which must be resolved using a clot remodeling scheme [16]. Another striking feature of the platelet aggregates that is evident from our 3D simulations is the asymmetry in thrombus morphology, despite a symmetric injury and initial velocity field. This asymmetry is a direct outcome of the stochastic nature of platelet deposition that is captured well by the LKMC module for tracking platelet trajectories and is intractable using a 2D approach. Furthermore, a 3D approach becomes necessary for tracking thrombus growth for a non-axisymmetric domain of interest. We demonstrated these advantages presented by the 3D model by carrying out simulations of thrombus growth in domains of clinical significance, namely straight and stenotic representations of blood vessels with a non-axisymmetric injury. While experimental data from cylindrical domains would be useful to validate the simulations, the fabrication of curvilinear microfluidic geometries with localized biocoatings is extremely difficult. Additionally, fluorescent imaging of cylindrical systems is challenging. However, the model parameters have been validated using simulations in the 3D rectangular channel. These parameters remain the same despite moving to a cylindrical simulation domain.

A number of key simplifications have been used to facilitate multiscale modeling of thrombus growth at a reasonable computational expense. Firstly, the use of a reduced coagulation cascade model to account for wall-derived TF represents a tractable but simple coarse-grained approach to reduce the computational cost associated with solving a set of ~50 simultaneous PDEs required to fully describe the full coagulation cascade. Moreover, the full coagulation cascade requires the specification of a large number of kinetic rate parameters, many of which are not precisely known in blood clots [35]. The reduced model, which predicted intra-clot thrombin levels that were validated against microfluidic perfusion experiments of whole blood over collagen/TF surfaces, was embedded into our multiscale framework at essentially zero computational expense. Additionally, to characterize the different platelet adhesion receptors that are operative during thrombus formation at different shear regimes, the basal rate of platelet attachment was augmented by an enhancement factor that was parametrized as a function of the local shear rate. Detailed descriptions of single-platelet capture would require adhesive dynamics calculations that would incur an extreme computational cost [36,37]. Therefore, this coarse-grained model that is based on an apparent rate of platelet attachment and detachment becomes a reasonable necessity.

The framework presented here may be further extended and employed in models for traumatic bleeding based on platelets from trauma patients [38]. In prior work, a lumped model of the cardiovascular system was coupled to a branching vasculature network model to predict the bleeding trajectory of a patient in response to defined injuries of severing blood vessels [39]. However, a coarse-grained hemostasis model that used a parametrized wound seal rate as a function of the shear rate was used in the study [40]. We expect that the present 3D simulation framework can be parameterized using platelets from trauma patients to enable the prediction of different patient bleeding trajectories (rate of bleeding and rate of hemostasis) following injury. This may help identify patients at risk for trauma-induced coagulopathy or guide transfusion decisions.

Computational modeling of thrombus growth has been studied extensively. Several of these studies make use of a continuum approach where platelets are treated as a chemical species that obeys the convection-diffusion-reaction equation for species transport [8–10,15]. This approach overcomes the significant computational burden associated with explicitly resolving individual blood cells and accounting for molecular-level interactions between these cells. However, certain key aspects are neglected, such as the ability to capture the stochastic nature of clot formation and embolization. The multiscale framework presented in this study falls under the class of hybrid models where blood velocity and platelet agonists are treated as continuum fields while platelets are resolved explicitly [7,13,17]. As a result, our framework has the ability to predict the structure of the clot at the resolution of an individual platelet at reasonable computational cost. Our framework is closest to the study by Yazdani et al. where platelets were treated as explicit particles and the role of VWF was included implicitly using a shear-dependent platelet adhesion model [17]. The strength of our framework is the ability to account for patient-specific platelet phenotypes by using NN models trained on calcium-trace data obtained from patient-specific multicomponent agonist exposure experiments to perform robust 3D simulations of thrombosis in geometries of clinical relevance. We are unaware of any 3D model of thrombosis to be capable of including a high-dimensional platelet phenotype. Consequently, the present model, when suitably trained, can predict the patient-specific effect of antiplatelet drug treatments and impacts of blood disorders such as von Willebrand disease in realistic 3D settings within the vasculature. This work therefore represents a critical step in exploring new opportunities for probing and understanding the mechanisms of heart attack and stroke, which require simulations of complex pulsatile flows in patient-specific coronary or carotid arterial geometries. The use of high-performance parallel computing to solve how clots grow in patient-derived, diseased coronary stenotic geometries is the subject of future work.

## Supporting information

S1 Appendix Supplemental Methods. Fig A in S1 Appendix—Reduced model of the coagulation cascade.
(DOCX)
