# Supplementary material for: A three-dimensional multiscale model for the prediction of thrombus growth under flow with single-platelet resolution
Source: PLoS Comput Biol. 2022 Jan 28;18(1):e1009850. doi: 10.1371/journal.pcbi.1009850 (PMC8827456; doi:10.1371/journal.pcbi.1009850)
Supplement: S1 Appendix — Fig A in S1 Appendix—Reduced model of the coagulation cascade. (DOCX) [file pcbi.1009850.s001.docx]

**S1 Appendix: Supplemental Methods**

**Reduced model of the coagulation cascade**

A reduced kinetic model of coagulation under flow was formulated by Chen and Diamond [1] to include extrinsic tenase/FIXase activity, intrinsic tenase activity, prothrombinase activity, feedback activation of FXIa by thrombin, fibrin generation, and thrombin binding to fibrin (Fig A) using measured Michaelis-Menten kinetic parameters (Table B). The reduced model employs various physical and biochemical features of clotting under flow that are supported by experimental measurement. For the reaction topology shown in Fig A, these assumptions result in a reduced clotting model with only 8 ODEs for 6 reactive species undergoing 7 reactions (Table B) and 2 fibrin sites for reversible binding of thrombin.

$\frac{d {TF}^{*}}{dt}=-k_{i,TF}\cdot{{TF}^{*}}for k_{i,TF}=ln(2)/180s$

$\frac{d Xa}{dt}=\alpha_{1}\cdot{TF}^{*}+\alpha_{3}\cdot IXa-k_{i}\cdot Xa$

$\frac{d IXa}{dt}=\alpha_{2}\cdot{TF}^{*}+\alpha_{7}\cdot XIa-k_{i}\cdot IXa$ $\frac{d XIa}{dt}=\eta_{6} {\cdot\alpha}_{6}\cdot IIa-k_{elute}\cdot XIa -k_{i}\cdot XIa for \eta_{6}=0.36$

$\frac{d Fibrin}{dt}={\eta_{5} \cdot\alpha}_{5}\cdot IIa for \eta_{5}=0.05$

$\frac{d IIa}{dt}={\eta_{4} \cdot\alpha}_{4}\cdot Xa-\left( \frac{d {}^{E}S}{dt}+\frac{d {}^{\gamma}S}{dt} \right)-k_{elute}\cdot IIa -k_{i}\cdot IIa for \eta_{4}=0.18$

$\frac{d {}^{E}S}{dt}={}^{E}{k_{f}}\cdot IIa\cdot\left( {}^{E}{\theta_{total}}-{}^{E}S \right)-{}^{E}{k_{r}}\cdot{}^{E}S$

$\frac{d {}^{\gamma}S}{dt}={}^{\gamma}{k_{f}}\cdot IIa\cdot\left( {}^{\gamma}{\theta_{total}}-{}^{\gamma}S \right)-{}^{\gamma}{k_{r}}\cdot{}^{\gamma}S$

where : ${}^{E}{\theta_{total}}=(1.6)\cdot fibrin$ and ${}^{\gamma}{\theta_{total}}=(0.3)\cdot fibrin$

This reduced model for blood clotting on a collagen/TF surface under flow uses 19 parameters, only 3 of which were adjusted to fit experimental data:

1. 7 kinetic coefficients (α_i_) based on measured kinetics and plasma zymogen levels (Table B)
2. 1 initial surface TF* level based on specified [TF]_o_ = 1 TF/μm^2^ and [FVIIa]/[FVII] = 0.01.
3. 3 binding parameters: ^E^K_D_, ^γ^K_D_, k_f_
4. 2 known stoichiometric coefficients: 1.6 E-sites/monomer, 0.3 γ’-sites/monomer
5. 1 elution rate: k_elute_ = ln(2)/2s for free species of thrombin and FXIa
6. 2 inhibition rates: k_i_ = ln(2)/60s for FXa, FIXa, FXIa, FIIa; k_i,TF_ = ln(2)/180s for TF*
7. 3 effectiveness factors (η_4_, η_5_, η_6_) ≠ 1, adjusted to fit experimental data.

A plot of the thrombin (IIa) concentration in the clot core as a function of time predicted by the model is shown in Fig B. The concentration of free thrombin is input into to the NN module to determine platelet activation states. For further details about the reduced coagulation cascade model, see Chen and Diamond [1].

**NN module**

Pairwise agonist scanning (PAS) was used to obtain calcium traces for apixaban-treated, diluted and calcium dye-loaded platelet rich plasma (PRP) stimulated with all single and pairwise combinations of six different agonists used at zero, low, medium and high concentrations (0, 0.1, 1 and 10 x EC_50_⁠). The agonists considered were ADP, U46619 (thromboxane mimetic), convulxin (collagen mimetic), thrombin, GSNO (NO donor) and iloprost (prostacyclin analog). NN training was then carried out using the NN Toolbox in the MATLAB software package to predict platelet calcium level for any agonist dose and combination. Input concentrations were mapped onto the values -1, -0.333, +0.333 and +1 corresponding to 0, 0.1,1 and 10 x EC_50_ levels of each input. The structure of the *NARX* (Nonlinear AutoRegressive network with eXogenous inputs) model utilized two-layer processing (eight nodes in the first layer and four nodes in the second layer) and a tapped delay line with 128 s of feedback to each layer (Fig C). The hyperbolic tangent transfer function was used in all of the processing layers and a linear transfer function was used in the output layer. Initial states of the feedback were set to 0 corresponding to the resting platelet. The NN predicted the calcium level for the next time step based on the feedback vector (1, 2, 4, 8, 16, 32, 64 and 128s prior to the current instant) and the current concentration input from the six agonists. The NN output with range 0 to 1 is linearly mapped to platelet intracellular calcium with range 100nM to 1μM. The time step in the NN is 1s, but to achieve greater time resolution in the multiscale model, the solution of the NN at some time t_1_ < t* < t_2_, was interpolated from the previous solution time (t_1_) of the NN and a predicted solution of the NN at the next time (t_2_) using the current system state. A NN-ensemble for PAS of 4 healthy donors predicted averaged calcium concentration output for human platelets exposed to any combinations of the six agonists. See Lee and Diamond for detailed information validating PAS-NN prediction of [Ca^2+^(t)] beyond the pairwise training set for trinary stimulations, 4–6 agonist stimulations and sequential agonist stimulations [2].

**LKMC module**

The input for LKMC is the rate database for all possible events in the system. The rate of a platelet diffusing one lattice space in any direction is $\Gamma_{D}=D_{platelet}/h_{LKMC}^{2}$. Within the framework of overdamped Langevin dynamics, the drift rate of a particle moving between adjacent lattice points is given by $\Gamma_{C}=\boldsymbol{v}\boldsymbol{\cdot}\boldsymbol{e}_{\boldsymbol{i}}/h_{LKMC}$, where ***v*** is the velocity of the particle and ***e***_i_ represents the unit vector along a particular lattice direction. To obtain LKMC expressions that account for platelet motion, we consider the forward and backward LKMC hopping rates denoted by *Γ_i+_* and *Γ_i-_* , respectively. These rates are required to satisfy:

$$\Gamma_{i+}-\Gamma_{i-}=\Gamma_{C}$$

and

$$\frac{\Gamma_{i+}}{\Gamma_{i-}}=\frac{\left| \boldsymbol{v}\boldsymbol{\cdot}\boldsymbol{e}_{\boldsymbol{i}} \right| h_{LKMC}}{D_{platelet}}=\omega$$

Solving for the forward and backwards rates using gives the expressions for the forward and backwards rate derived by Lee and Sinno [3]:

$$\Gamma_{i+}=\Gamma_{C}\frac{\exp\left( \omega\right)}{\exp\left( \omega\right)-1}$$

$$\Gamma_{i-}=\Gamma_{C}\frac{1}{\exp\left( \omega\right)-1}$$

A binding event is only allowed if there is connectivity to the surface through one of the platelets. This rule prevents free-flowing platelet aggregates, which are not expected to significantly contribute to the surface-adhered platelet deposit over the length and time scales of interest.

With the specified rate database at system time *t,* LKMC chooses the time step of the next event as

$$\tau= -\frac{ln\left( u \right)}{\Gamma_{total}}$$

where *Γ_total_* is the total rate of all events in the system and *u* is a random number drawn from the uniform distribution in the interval *(0,1]*. The probability that event *i* with rate *Γ_i_* will be the next event is

$$P_{i}=\frac{\Gamma_{i}}{\Gamma_{total}}$$

For all simulations presented in this work, we used a lattice spacing of *h_LKMC_* = 1μm.

**LB module**

The lattice Boltzmann (LB) method is used to solve for the velocity profile described by the Navier-Stokes and continuity equations defined in Equations 1-2. LB indirectly solves these equations by simulating the streaming and collision of fluid particles during one time step on a uniform lattice. In this case for a 3D lattice, each lattice node streams particles to its 19 nearest neighbors and itself (D3Q19 lattice). After streaming, the particle distribution at each lattice site is relaxed to an equilibrium configuration. The macroscopic velocity profile is obtained from moments of the particle distribution at each node. The lattice spacing was defined as *h_LB_* = 1.5μm, and the time step was set at *Δt* = 10^-7^s. The walls of the domain and aggregated platelets defined the no-slip boundary conditions.

The open-source LB solver Palabos was used to carry out all computations of the velocity and shear rate [4]. The simulation domain (microfluidic device, cylinder, or stenosis) was provided as a stereolithography (STL) file to Palabos, which then used the description of the geometry provided in the STL file to create a uniform hexahedral LB lattice. The STL files for each case were generated using the free software package Salome [5].

**FVM module**

The convection-diffusion-reaction equation, as defined in Equation 15, was solved with the Finite Volume Method (FVM). The open-source software package OpenFOAM was used to carry out our FVM computations of spatiotemporal agonist concentration profiles [6]. The snappyHexMesh utility in OpenFOAM was used to generate the initial cubic FVM mesh from an STL description of the geometry generated by Salome. An important feature of thrombus growth under flow is that the clotting region, especially at early times, occupies only a small portion of the computational domain. Consequently, using a uniform mesh throughout the entire domain incurs a large computational cost where many mesh nodes must be applied to achieve a reasonable resolution around each platelet. An adaptive meshing scheme was designed to allow the mesh density to be refined in the clotting region of an evolving rough clot surface. Starting from an initial mesh spacing of 3μm, the dynamicRefineFvMesh utility provided by OpenFOAM was used to perform topological refinements to the mesh. At mesh locations where platelets become sufficiently activated for dense granule release, the mesh was refined to 0.75μm. In such a scenario, a single platelet overlapped several FVM cells, so the cell at the center of the activated platelet was treated as the source element for the PDE calculation. The time derivative was approximated using the implicit Crank Nicolson scheme with a time step of 0.01s.

**Module integration**

The flow of information between models is given in Fig 1B. LKMC provides the position of all platelets in the domain and the bonding state of each platelet. LKMC requires the velocity field of the fluid (LB) to calculate convective rates of motion and the activation state of each platelet (NN) to determine the bonding and unbonding rates. The NN provides the activation state of each platelet, and the input into the NN is the local concentration of platelet agonists, which requires both the platelet positions (LKMC) and the concentration field of soluble agonists (FVM). The LB method provides the velocity field and requires the location of all bonded platelets for the location of the no-slip surfaces. FVM provides the concentration field and requires the release rate of platelets, which depends on the location of platelets (LKMC) and the activation states (NN), and the velocity field (LB).

The coupling of the individual models only occurred after a time interval of every 0.1s. At the start of the simulation LKMC, LB, FVM, and the NN were all specified by the initial condition for each method. Each method was stepped forward in time until the first coupling time was reached. During this update, all modules share information: LKMC updated the positions of all platelets and the bound states of all platelets in FVM, LB, and NN; LB updated the velocity field in LKMC and FVM; FVM updated the concentration field in NN; and NN updated the activation state in FVM and LKMC. This process repeated until the end of the simulation time. The multiscale model is composed of several modules that require the exchange of information between each other, with individual modules each having their respective open-source software packages. To facilitate the coupling between different modules at each coupling time, we use the Multiscale Universal Interface (MUI) [7]. MUI is a lightweight library that helps achieve data exchange between each module with minimal modifications to individual module source codes.

The time scale for velocity field relaxation was generally << 0.01s, so the velocity field reached a steady state in LB significantly before the next update time. To gain computational efficiency, LB was only simulated until a steady-state was achieved.

**Supplemental References**

1. Chen J, Diamond SL. Reduced model to predict thrombin and fibrin during thrombosis on collagen/tissue factor under venous flow: Roles of γ’-fibrin and factor XIa. PLOS Comput Biol. 2019;15: e1007266. Available: https://doi.org/10.1371/journal.pcbi.1007266

2. Lee MY, Diamond SL. A human platelet calcium calculator trained by pairwise agonist scanning. PLoS Comput Biol. 2015;11: e1004118.

3. Lee YK, Sinno T. Analysis of the lattice kinetic Monte Carlo method in systems with external fields. J Chem Phys. 2016;145: 234104.

4. Latt J, Malaspinas O, Kontaxakis D, Parmigiani A, Lagrava D, Brogi F, et al. Palabos: parallel lattice Boltzmann solver. Comput Math with Appl. 2021;81: 334–350.

5. David G, Chevalier T, Meunier G. Unification of physical data models. Application in a platform for numerical simulation: SALOME. 2006 12th Biennial IEEE Conference on Electromagnetic Field Computation. IEEE; 2006. p. 228.

6. Jasak H, Jemcov A, Tukovic Z. OpenFOAM: A C++ library for complex physics simulations. International workshop on coupled methods in numerical dynamics. IUC Dubrovnik Croatia; 2007. pp. 1–20.

7. Tang Y-H, Kudo S, Bian X, Li Z, Karniadakis GE. Multiscale universal interface: a concurrent framework for coupling heterogeneous solvers. J Comput Phys. 2015;297: 13–31.

8. Giles C. The platelet count and mean platelet volume. Br J Haematol. 1981;48: 31–37.

9. Trudnowski RJ, Rico RC. Specific gravity of blood and plasma at 4 and 37 C. Clin Chem. 1974;20: 615–616.

10. Wells RE, Merrill EW. Influence of flow properties of blood upon viscosity-hematocrit relationships. J Clin Invest. 1962;41: 1591–1598.

11. Paulus J-M. Platelet size in man. 1975.

12. Nanne EE, Aucoin CP, Leonard EF. Shear rate and hematocrit effects on the apparent diffusivity of urea in suspensions of bovine erythrocytes. ASAIO J (American Soc Artif Intern Organs 1992). 2010;56: 151.

13. Hubbell JA, McIntire L V. Platelet active concentration profiles near growing thrombi. A mathematical consideration. Biophys J. 1986;50: 937–945.

14. Holmsen H, Storm E, Day HJ. Determination of ATP and ADP in blood platelets: a modification of the firefly luciferase assay for plasma. Anal Biochem. 1972;46: 489–501.

15. De Caterina R, Giannessi D, Gazzetti P, Bernini W. Thromboxane-B2 generation during ex-vivo platelet aggregation. J Nucl Med Allied Sci. 1984;28: 185–196.

16. Beigi R, Kobatake E, Aizawa M, Dubyak GR. Detection of local ATP release from activated platelets using cell surface-attached firefly luciferase. Am J Physiol Physiol. 1999;276: C267–C278.

**Table A.** List of parameters and parameter values used in the multiscale model.

| **Symbol** | **Name** | **Value** | **Ref./Comment** |
| --- | --- | --- | --- |
| N_platelet_ | Platelet count | 1.5 x 10^5^ µL^-1^ | [8] |
| ρ_blood_ | Density of blood | 1000 kg/m^3^ | [9] |
| µ_blood_ | Viscosity of blood | 3 cP | [10] |
| R_platelet_ | Radius of platelet | 1.5 µm | [11] |
| D_platelet_ | Diffusion coefficient of platelet | 1.25 x 10^-7^ cm^2^/s | [12] |
| D_ADP_ | Diffusion coefficient of ADP | 2.37 x 10^-6^ cm^2^/s | [13] |
| D_TXA2_ | Diffusion coefficient of TXA_2_ | 2.14 x 10^-6^ cm^2^/s | [13] |
| M_ADP_ | Total amount of released ADP | 2.5 x 10^-8^ nmol/platelet | [14] |
| M_TXA2_ | Total amount of released TXA_2_ | 1 x 10^-9^ nmol/platelet | [15] |
| τ_ADP_ | Characteristic release time for ADP | 5 s | [16] |
| τ_TXA2_ | Characteristic release time for TXA_2_ | 100 s | [15] |
| C_CVX_ | Effective concentration of collagen (in units of CVX) | 5 x EC_50_ | Soluble vs surface ligand |
| **Estimated parameters** |  |  |  |
| $\alpha_{min}$ | Range of integrin activation | 0.001 | ~ 1 active integrin per 1000 on a resting platelet |
| $\alpha_{max}$ |  | 1 |  |
| $\alpha_{min,amp}$ | Amplification of transient calcium integral | 1 |  |
| $\alpha_{max,amp}$ |  | 50 |  |
| n | Sharpness of activation function for total calcium integral | 0.75 | Expected range:  0.5 < n < 2 |
| n_amp_ | Sharpness of activation function for transient calcium integral | 2 |  |
| ξ_50_ | Critical value for 50% platelet activation and dense granule release | 9 µM-s | Strong calcium mobilization (e.g., 1 μM persisting for 9s) |
| $k_{att}^{collagen}$ | Attachment rate constant for platelet-collagen binding | 500 s^-1^ |  |
| $k_{att}^{platelet}$ | Attachment rate constant for platelet-platelet binding | 50 s^-1^ |  |
| $k_{det}^{collagen}$ | Detachment rate constant for platelet-collagen binding | 1 x 10^-5^ s^-1^ |  |
| $k_{det}^{platelet}$ | Detachment rate constant for platelet-platelet binding | 1 x 10^-4^ s^-1^ |  |
| γ_c_ | Critical shear rate | 200 s^-1^ |  |
| γ_c_’ |  | 50,000 s^-1^ |  |

**Table B.** Reactions and kinetic parameters used in the ODEs model. Simplified clotting reactions neglecting limits in activated cofactor generation, plasma zymogen concentrations, and kinetic parameters of coagulation where η is the effectiveness factor (actual rate with transport limits/theoretical maximum rate). For each reaction, α_0_ = k_cat_ [S]_0_/(K_m_+[S]_0_). = k_cat_[S]_0_/(Km+[S]_0_). Reversible binding of thrombin to the weak and strong site in fibrin was treated as kinetically-controlled, reversible adsorption.

| **Reactions** | **Enzyme** | **[S]_0_** | **k_cat_**  **(s^-1^)** | **K_m_**  **(µM)** | **α**  **(s^-1^)** | **η** |
| --- | --- | --- | --- | --- | --- | --- |
| $X\underset{\to}{\mathrm{TF}^{*}}\mathrm{Xa}$ | TF/VIIa | X_0_=0.17 µM | 1.15 | 0.24 | 0.46 | 1 |
| $\mathrm{IX}\underset{\to}{\mathrm{TF}^{*}}\mathrm{IXa}$ | TF/VIIa | IX_0_=0.09 µM | 1.8 | 0.42 | 0.32 | 1 |
| $X\underset{\to}{\mathrm{IXa}}\mathrm{Xa}$ | IXa/VIIIa | X_0_=0.17 µM | 8.2 | 0.082 | 5.42 | 1 |
| $\mathrm{II}\underset{\to}{\mathrm{Xa}}\mathrm{IIa}$ | Xa/Va | II_0_=1.4 µM | 30 | 0.3 | 24.7 | 0.18 |
| $\alpha-\mathrm{fbg}\underset{\to}{\mathrm{IIa}}desA-Fn1+FPA$ | IIa | α-fbg_0_=18 µM | 80 | 6.5 | 5.88 | 0.05 |
| $\mathrm{XI}\underset{\to}{\mathrm{IIa}}\mathrm{XIa}$ | IIa/p* | XI_0_=31 nM | 1.3x10^-4^ | 0.05 | 4.98x10^-5^ | 0.36 |
| $\mathrm{IX}\underset{\to}{\mathrm{XIa}}\mathrm{IXa}$ | XIa/p* | IX_0_=0.09 µM | 0.21 | 0.2 | 0.065 | 1 |
| **Thrombin binding to fibrin** | | | **K_d_**  **(µM)** | **k_f_**  **(µM^-1^s^-1^)** | **k_f_**  **(s^-1^)** |  |
| $IIa+E site\underset{\leftrightarrow}{}IIa\cdot E site$ | | | 2.8 | 100 | 280 |  |
| $IIa+\gamma site\underset{\leftrightarrow}{}IIa\cdot\gamma site$ | | | 0.1 | 100 | 10 |  |

**Fig A. Reduced model of the coagulation cascade.** A simplified ODEs model of the coagulation cascade shown here was used to determine the concentration of free thrombin within the thrombus core. All zymogens were assumed to enter the clot core by diffusion to maintain their plasma level [S]_o_. All active enzymes had a 1-minute half-life, with TF* set to 3 min (since FVIIa generation was ignored). Free thrombin and FXIa eluted by diffusion from the core with a 2s half-life. The core thickness was set to 15μm, with 50% of platelets by vol. Only the activated proteases are shown for simplicity. The concentration of free thrombin predicted by the reduced model was used an input to the NN module.

**
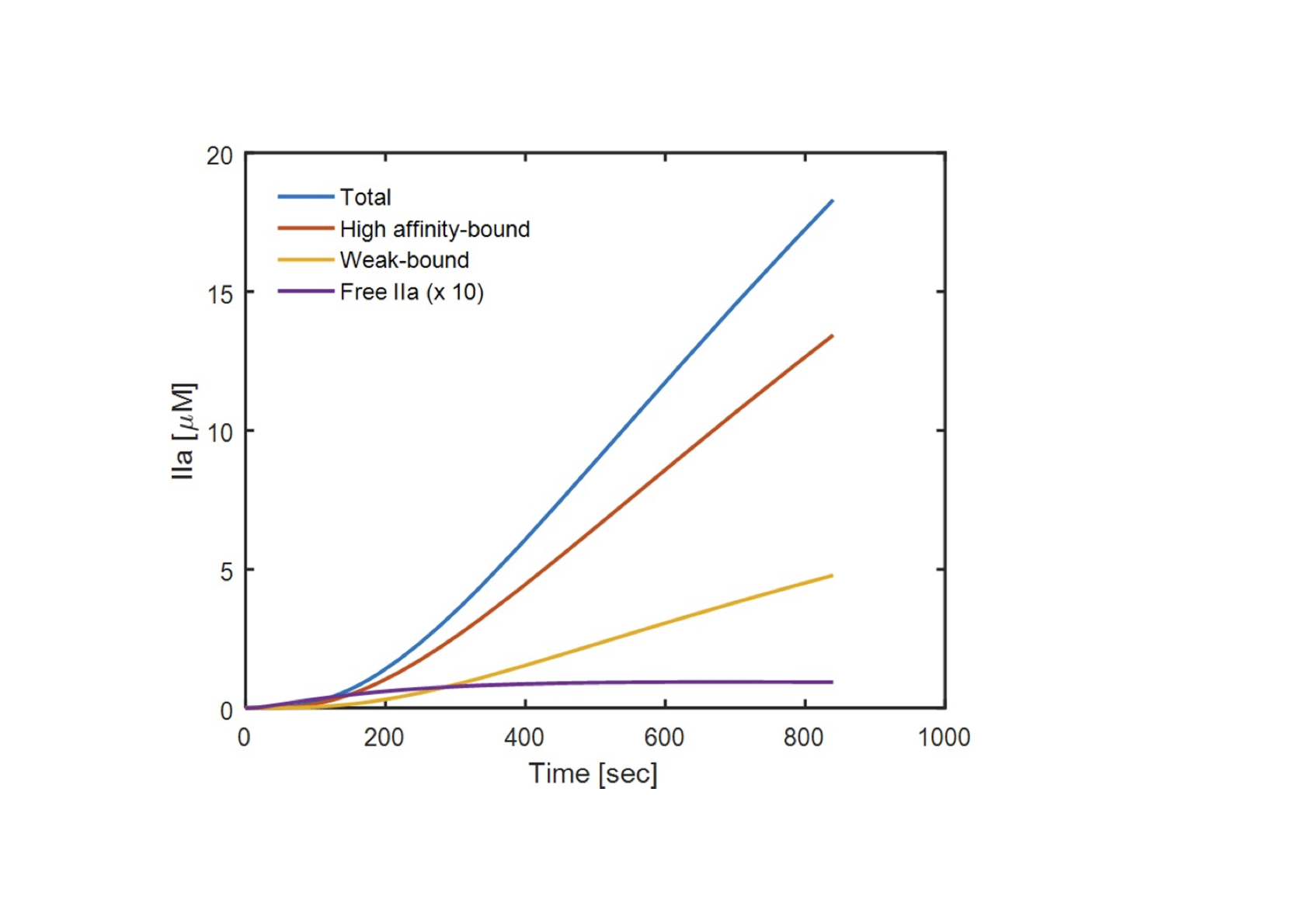
**

**Fig B. Concentration of thrombin predicted by the reduced coagulation cascade model**. The majority of intra-thrombus thrombin is bound to the γ’-site in fibrin with <100 nM as free thrombin. The concentration of free thrombin predicted by the reduced model is used an input to the NN module to determine intra-platelet calcium ion concentration. The free thrombin concentration predicted by the model is within the dynamic range (< 10 EC_50_).


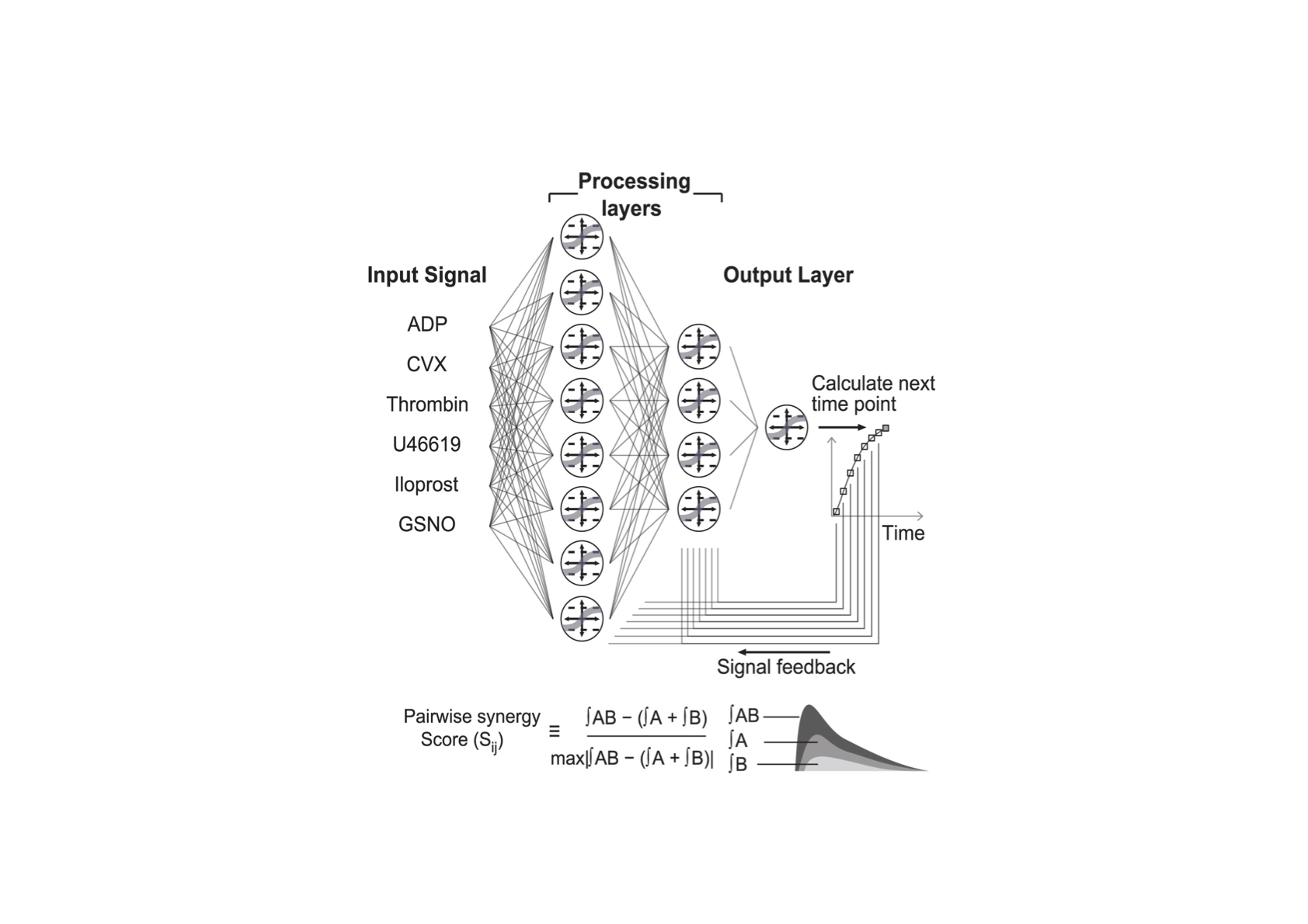


**Fig C. Schematic of the NN architecture**. A 2-layer, 12-node NN architecture was employed for prediction of intraplatelet calcium in response to multicomponent agonist exposure.
